# Against the Resilience of High-Grade Gliomas: Gene Therapies (Part II)

**DOI:** 10.3390/brainsci11080976

**Published:** 2021-07-23

**Authors:** Alice Giotta Lucifero, Sabino Luzzi

**Affiliations:** 1 Neurosurgery Unit, Department of Clinical-Surgical, Diagnostic and Pediatric Sciences, University of Pavia, 27100 Pavia, Italy; alicelucifero@gmail.com; 2 Neurosurgery Unit, Department of Surgical Sciences, Fondazione IRCCS Policlinico San Matteo, 27100 Pavia, Italy

**Keywords:** gene therapy, glioblastoma, immunomodulation, malignant brain tumor, oncolytic viruses, suicide genes, target gene, tumor suppressor genes

## Abstract

Introduction: High-grade gliomas (HGGs) still have a high rate of recurrence and lethality. Gene therapies were projected to overcome the therapeutic resilience of HGGs, due to the intrinsic genetic heterogenicity and immune evasion pathways. The present literature review strives to provide an updated overview of the novel gene therapies for HGGs treatment, highlighting evidence from clinical trials, molecular mechanisms, and future perspectives. Methods: An extensive literature review was conducted through PubMed/Medline and ClinicalTrials.gov databases, using the keywords “high-grade glioma,” “glioblastoma,” and “malignant brain tumor”, combined with “gene therapy,” “oncolytic viruses,” “suicide gene therapies,” “tumor suppressor genes,” “immunomodulatory genes,” and “gene target therapies”. Only articles in English and published in the last 15 years were chosen, further screened based on best relevance. Data were analyzed and described according to the PRISMA guidelines. Results: Viruses were the most vehicles employed for their feasibility and transduction efficiency. Apart from liposomes, other viral vehicles remain largely still experimental. Oncolytic viruses and suicide gene therapies proved great results in phase I, II preclinical, and clinical trials. Tumor suppressor, immunomodulatory, and target genes were widely tested, showing encouraging results especially for recurrent HGGs. Conclusions: Oncolytic virotherapy and suicide genes strategies are valuable second-line treatment options for relapsing HGGs. Immunomodulatory approaches, tumor suppressor, and target genes therapies may implement and upgrade standard chemoradiotherapy. Future research aims to improve safety profile and prolonging therapeutic effectiveness. Further clinical trials are needed to assess the efficacy of gene-based therapies.

## 1. Introduction

High-grade gliomas (HGGs) are deadly brain tumors accounting for 70% of all central nervous system neoplasms [1,2,3], and the optimization of their management is among the most demanding challenging of the modern neuro-oncology. The reasons for their resilience toward treatment strategies depend on the high cell turnover, pathological neoangiogenesis, and genetic landscape heterogenicity [4,5,6,7,8,9,10,11]. Established guidelines include gross total surgical resection followed by adjuvant chemoradiotherapy [12,13]. In the effort to improve the prognosis of these tumors, characterized by a median survival of only 12–15 months [14,15,16,17], those treatment options considered as “standard of care” have been recently augmented with newer tailored and immune-based technologies. Recent advances in genetic, nanotechnologies, biotechnologies, and translational medicine provided the means for the development of more sophisticated approaches, including gene therapies which have polarized growing attention during the last few years [18,19,20,21,22,23,24,25,26].

Gene therapies aim to transfer manipulated genetic payload to glioma cells via engineered vectors resulting in genome rearrangement, oncolysis, and tumor regression [27,28,29,30,31,32,33]. The goal of the present study is to overview the landscape of gene therapies for HGGs focusing on vectors’ engineering, oncolytic mechanisms, and clinical trials. Limitations and future perspectives of the gene-based approach are also discussed.

## 2. Methods

The Preferred Reporting Items for Systematic Reviews and Meta-Analyses (PRISMA) guidelines were used to perform a comprehensive online systematic literature review. PubMed/Medline (https://pubmed.ncbi.nlm.nih.gov, accessed on 1 April 2021) and ClinicalTrials.gov (https://clinicaltrials.gov, accessed on 30 January 2021) databases were employed, combined with Medical Subject Headings (MeSH) terms and words text. The MeSH terms and keywords were “high-grade glioma,” “glioblastoma,” and “malignant brain tumor”, merged with “gene therapy,” “oncolytic viruses,” “suicide gene therapies,” “tumor suppressor gene therapies,” “immunomodulatory gene therapies,” and “gene target therapies” to limit the research to the innovative therapies of gene delivery.

English language, or translated, and publication date back to the last 15 years were the eligibility criteria. Systematic reviews and editorials were included and further screened based on the best match and relevance.

On the ClinicalTrials.gov database, the search words were “high-grade glioma”, “glioblastoma”, “malignant brain tumor”, “gene therapies”, and “oncolytic viruses”. Interventional studies and clinical trials were chosen without restrictions for phase or recruitment status. Studies about gene therapies for malignant brain gliomas were finally selected. An overview of the classification criteria, vectors, therapeutic principles, and pharmacological agents was conducted.

The inclusion and exclusion criteria applied in the systematic literature review are described in Table 1.

## 3. Results

**Figure 1:**
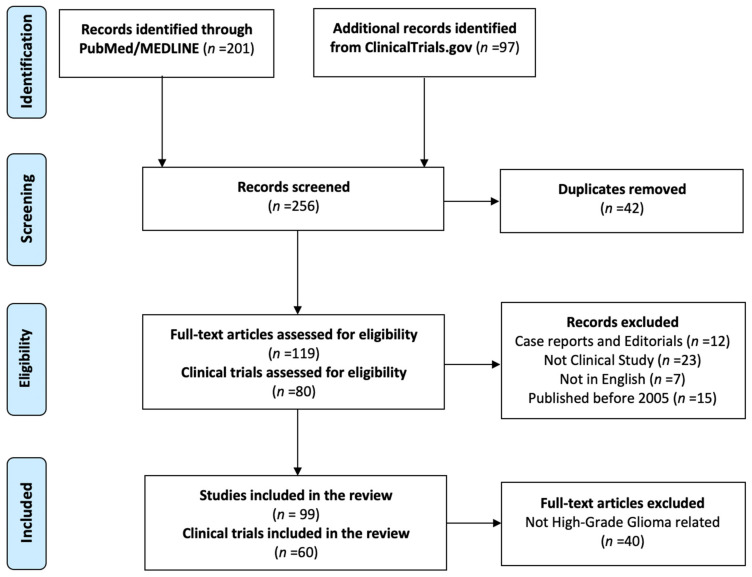
PRISMA flow-chart for systematic review.

The review returned a total of 201 articles and 97 clinical trials. After duplicates removal and application of the exclusion criteria, a total of 99 articles and 60 clinical trials were considered for eligibility. Figure 1 shows the PRISMA flow chart.

### 3.1. Vectors

The gene therapy feasibility is guaranteed by engineered vectors, viral or non-viral, able to deliver genetic material into target cells [30,31]. Viral vectors are non-toxic purified viruses designed to transfer genetic payload without triggering the infection [34]. Two types of viral carriers are now clinically approved: the replication-competent and -incompetent viruses. The formers are mutated viral agents which maintain the self-replication ability, transinfect tumor cells inducing lysis. The replication-incompetent ones are genome-deleted viruses with reduced toxicity [35,36].

Recently advanced nanotechnologies made possible the design of nanoparticles, micron-sized molecules, suitable as non-viral carriers with low toxicity and immunogenicity [37]. Nanoparticles include liposomes and polymers, which pass through the blood-brain barrier and cross the tumor cell membrane via endocytosis. Liposomes are lipid vesicles which load electrostatically DNA and RNA plasmid and transfer genes to target cells. Polymers are macromolecules which directly bind DNA and include nucleotides into the tumor cells genome. The polyethyleneimine (PEI), a linear polymer, was widely tested, frequently combined with polyethylene glycol (PEG) or β-cyclodextrin to improve biodistribution and increasing tumor targeting [38,39,40,41]. The polymer polyamidoamine (PAMAM) was also employed in delivering therapeutic genes to glioma cells [42,43].

Moreover, iron oxide nanoparticles (SPIONs), enriched with PEG or PEI, were used as genetic carriers, allowing them to be displayed in magnetic resonance imaging [44,45]. Table 2 presents a comparison between viral and non-viral carriers.

### 3.2. Classification of Gene Therapies for High-Grade Glioma

Gene therapies can be categorized based on molecular mechanisms, carriers involved, and therapeutic gene transferred. The most promising strategies include the replication-competent oncolytic viruses (OVs), suicide gene therapy, tumor suppressor gene delivery, immunomodulatory strategies, and gene target therapies. Table 3 reports the classification of gene therapies for HGGs.

#### 3.2.1. Oncolytic Virotherapy

**Figure 2:**
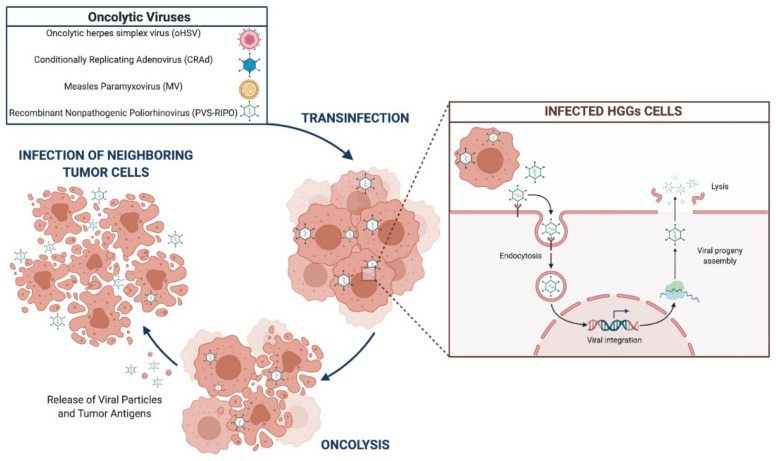
Oncolytic viruses’ mechanism of action.

OVs are designed to selectively infect tumor cells, self-replicate, and induce apoptosis [46,47,48]. Oncolytic herpes simplex virus (oHSV), conditionally replicating adenovirus (CRAd), measles paramyxovirus (MV), and recombinant non-pathogenic poliorhinovirus (PVS-RIPO) are under evaluation for HGGs treatment (Figure 2).

##### oHSVs

oHSVs are double-stranded DNA viruses, attenuated through the inactivation of the unique long (UL) 39 gene, which encodes the ribonucleotide reductase (ICP6), and the deletion of protein synthesis-promoting factors (γ34.5) [49,50].

The oHSV1716, the first generation oHSV, was devoid of both γ34.5 copies. oHSV1716 was tested in several clinical trials, showing good results as an adjuvant agent for HGGs treatment. In 2000, Rampling et al. evaluated the toxicity of oHSV1716 after intratumoral inoculation. They treated nine patients affected by relapsing HGGs showing a good safety profile [51]. In 2002, Papanastassiou et al. administered 1 × 10^5^ plaque-forming units (PFUs) of oHSV1716 to 12 patients with recurrent HGGs and, 9 days after the inoculation, tumors were surgically removed. Histological findings demonstrated the active intratumoral viral replication [52]. oHSV1716 was also employed in phase II clinical trial for HGGs treatment, combined with dexamethasone and surgery (#NCT02031965). Despite the good tolerance, the major weakness of this strategy lies in the deletion of γ34.5, which reduces viral activity and efficacy [53].

The oHSVG207, deleted γ34.5 and inactivated ICP6, was employed in a phase I clinical trial which reported radiological evidence of antitumor activity in 21 patients and an excellent safety profile (dose 3 × 10^9^ PFU) [54]. Nine years later, the same group tested the injection of oHSVG207 directly in surgical cavities after surgery, as adjuvant therapy. Histology confirms viral replication activity and radiologic evidence proved the antitumor activity [55]. Several phases I, and I/II clinical trials employed the HSVG207, locally administered, as a single agent or in combination with radiation therapy. Results showed few side effects, a synergic effect with concurrent radiotherapy, but the efficacy is still limited (#NCT00028158, # NCT03911388, #NCT00157703, # NCT02457845).

The mutant HSV (rQNestin34.5) and M032, new generation oHSVs, were tested in two phases I clinical trials, showing relevant oncolytic activity against HGGs (#NCT03152318, # NCT02062827).

##### CRAd

CRAds are non-enveloped DNA adenovirus engineered by the removal of E1A-B genes, which inhibit the binding to the retinoblastoma protein (pRB) and p53, respectively, and block infected cell apoptosis.

The ONYX-015, modified with deletion of E1B genes, selectively targets tumor cells with aberrant p53 pathways [56]. A phase I clinical study employed the ONYX-015 with a dose-escalation protocol. It was injected into the surgical cavity after removal of 24 HGGs (dose from 1 × 10^7^ to 1 × 10^10^ PFUs). No side effects were registered, but the progression-free survival (PFS) rate was only 46 days, and overall survival (OS) of 6 months [57]. A phase I clinical trial evaluated the combination of ONYX-015 with cisplatin and fluorouracil, as adjuvant therapy after surgical removal (#NCT00006106). The study showed good tolerance to the OVs, but the treatment efficacy was still not significant.

DNX-2401 (Ad5Delta24) deleted in the E1A gene, selectively target glioma cells harboring pRb pathways mutations [58]. In 2018, Lang and colleagues treated 37 patients with relapsed HGGs with intratumoral injection of DNX-2401. Patients were stratified into two groups, the first received a single dose, the second was treated by resection followed by the inoculation of DNX-2401 in the surgical cavity. The median OS was of 9.5 months and 13.0 months for the group 1 and 2, respectively (#NCT00805376) [59]. Several phase I trials tested the intratumoral inoculation of DNX-2401 with temozolomide (#NCT01956734), interferon-γ (INFγ) (#NCT02197169). In 2017, at the American Society of Clinical Oncology (ASCO) Annual Meeting I, Lang and colleagues presented the results of their clinical study (#NCT02197169) on 27 enrolled patients affected by recurrent HGGs, of which nine were treated with DNX-2401 as monotherapy and 18 with DNX-2401 and INFγ. The 12-month OS was 33% and 18-month was OS was 22% in both groups, independently from the type of treatment.

Despite encouraging results in volumetric tumor reduction with single DNX-2401/INFγ administration, no significant difference in survival was reported between the two groups [60].

An active phase II trial is studying the combination of intratumoral DNX-2401 and adjuvant systemic pembrolizumab for HGGs treatment of 49 patients with malignant brain tumors (#NCT02798406). An ongoing phase I trial is evaluating the efficacy of DNX-2401 after conventional surgery (#NCT03896568).

DNX-2440, the mutant variant of DNX-2401, was engineered with the insertion of the OX40 ligand gene. The OX40, expressed on glioma cells, boosts the antitumoral immune response. DNX-2440 is currently under evaluation for HGGs treatment (#NCT03714334).

##### MV

MV, an enveloped RNA virus, exhibits the mutated hemagglutinin envelope glycoprotein H, also known as Edmonston strain, which selectively targets the CD46 on glioma cells [61,62]. MV was engineered to express the circulating carcinogenic embryonic antigen (CEA), useful to assess the virus replication and oncolytic activity [63]. A phase I study tested the toxicity of MV-CEA association and no severe side effects were reported (#NCT00390299) [64]. MV was also designed to express interleukin-13 (IL-13) directed to the IL-13Rα2 receptor on glioblastoma (GBM) cells, or the single-chain antibody versus the vIII variant of epidermal growth factor receptor (EGFRvIII) [65,66,67].

##### PVS-RIPO

PVS-RIPO is an attenuated Sabin poliovirus engineered by the replacement of the internal ribosomal entry site (IRES) with the IRES from a human rhinovirus, to reduce the viral neuropathogenicity [68,69,70]. The tropism of PVS-RIPO for tumor cells is determined by the poliovirus receptor CD155, expressed on HGGs cells [71,72].

PVS-RIPO was tested for treatment of pediatric (#NCT03043391) and adult recurrent HGGs as monotherapy, in combination with a single-cycle of lomustine (#NCT02986178), or with the anti-PDL1 antibody atezolizumab (NCT03973879). Results from the aforementioned clinical trials showed a sufficient anticancer efficacy, but a low safety profile.

Table 4 reports a comprehensive summary of clinical trials on oncolytic virotherapy for HGGs.

#### 3.2.2. Suicide Gene Therapies

**Figure 3:**
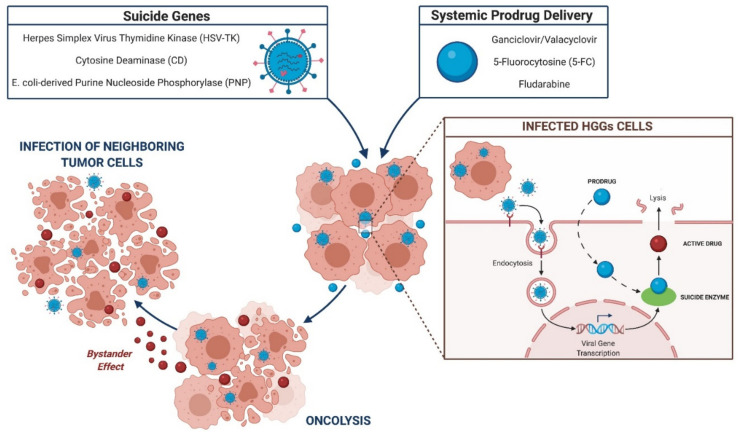
Suicide gene mechanism of action.

The suicide gene strategy is grounded on the viral transfer of “suicide genes” to target cells, which encode for enzymes able to convert prodrug to active compound [73,74]. The inactive prodrug is administered systematically and activated at the tumor site by the suicide enzymes, resulting in oncolytic effect and tumor cell apoptosis [75]. For HGGs treatment, the suicide transgene evaluated in clinical and preclinical studies are as follows: herpes simplex virus thymidine kinase (HSV-TK), cytosine deaminase (CD), and *E. coli*-derived purine nucleoside phosphorylase (PNP) (Figure 3).

##### HSV-TK

The HSV-TK enzyme catalyzes the monophosphorylation of the ganciclovir/valacyclovir, which is after triphosphorylates and activates intracellular kinases. The active drug blocks the S-phase and arrests the cell circle, leading to inhibition of DNA synthesis and tumor lysis [76,77,78,79].

In 2000, the phase III clinical study piloted by Rainov and colleagues tested the effect of HSV-TK gene therapy for 248 patients with newly diagnosed HGGs. Patients received an intratumoral inoculation of retroviral HSV-TK, followed by standard surgery, radiation therapy, and systemic administration of ganciclovir for 2 weeks. The study group was compared to the control one (conventional surgery and radiotherapy) and not significant differences in PFS and OS were founded [80]. Two recruiting phase I/II clinical trials tested the HSV-TK combined with replication-defective adenoviral vector (ADV/HSV-TK) and administered with valacyclovir (#NCT03603405, #NCT03596086). Results demonstrated the safety of this strategy with promising antitumoral efficacy. In 2000, Sandmair et al. conducted a phase I clinical trial intending to prove the efficacy and transinfection efficiency of replication-defective retrovirus or adenovirus-mediated HSV-TK/ganciclovir. 21 patients with newly diagnosed or recurrent HGGs were recruited, divided in two groups, and treated with retrovirus or adenoviruses. The OS of the adenovirus-mediated strategy was much higher [81]. Germano and colleagues tested the same ADV/HSV-TK strategy in a phase I trial and results showed a PFS of 112 weeks and an OS of 248 weeks [82]. In 2008, the ASPECT phase III clinical trial studied the ADV/HSV-TK for HGGs treatment. Out of 236 patients recruited who underwent surgical resection, 119 were randomized for the inoculation of ADV/HSV-TK locally at the tumor cavity, followed by systemic ganciclovir for two weeks. The PFS rates were 268 days and 308 days, and OS were 452 days and 497 days for the study group compared to the control one, respectively [83]. In 2016, Wheeler et al. enrolled 48 patients, harboring newly diagnosed HGGs, treated with ADV/HSV-TK and postoperative intravenously valacyclovir. This group was compared to the control one, treated with conventional surgery and adjuvant chemoradiotherapy. The PFS rate was 8 months for the study group and 6.5 months for the control one; the OS was 17 versus 13.5 months for the study and the control group, respectively (#NCT00589875). Results demonstrated the greatest effectiveness in the use of AD as the carrier for HSV-TK gene therapy.

An active phase I trial is evaluating a combined innovative approach, exploiting ADV/HSV-TK and the prodrug valacyclovir, associated with a checkpoint inhibitor (nivolumab), chemotherapy (temozolomide), and conventional radiation, to evaluate the safety profile and achievability of this enhanced strategy (#NCT03576612).

##### CD

The bacterial enzyme CD catalyzes the activation of the prodrug 5-fluorocytosine (5-FC) in oncolytic 5-fluorouracil (5-FU), selectively in glioma cells [28,84,85]. Toca 511, a replication-competent retrovirus, loads the CD and transinfect tumor cells. It promotes the expression of CD, which active the 5-FU, able to irreversibly blocks DNA synthesis and leads to cell apoptosis [86].

From 2016, Cloughesy and his group employed the Toca 511/5-FC in two clinical trials. The first one (#NCT01156584) tested the Toca 511 via stereotactic transcranial injection or intravenously injection [87]. In the subsequent study (#NCT01470794), they administered Toca 511 in the surgical cavity with a subsequent administration of Toca FC antifungal agent. Patients affected by recurrent or progressive HGGs were enrolled, and results showed a good safety profile and a median OS of 12–14 months [86]. Despite these initial encouraging results, Cloughesy et al. 2019 designed a phase III study which reported the therapeutic failure of Toca 511/5-FC, compared to the standard of care, in 271 patients with recurrent HGGs (#NCT02414165).

##### PNP

The *E. coli*-derived PNP converts adenosine ribonucleosides, as the fludarabine, in the active adenine compound, namely the 2-fluoroadenine. It interferes with RNA replication and cell cycle [88,89]. PNP, delivered with HSV or retrovirus vehicles, showed good long-term efficacy in preclinical models for malignant tumors treatment [90,91,92,93,94].

Moreover, the co-administration of antibiotic therapy, which suppresses the intestinal flora, could overactivated the PNP gene therapy intensifying the prodrug conversion [95,96]. Table 5 reports a comprehensive summary of clinical trials on suicide gene therapies for HGGs.

#### 3.2.3. Tumor Suppressor Gene Therapies

**Figure 4:**
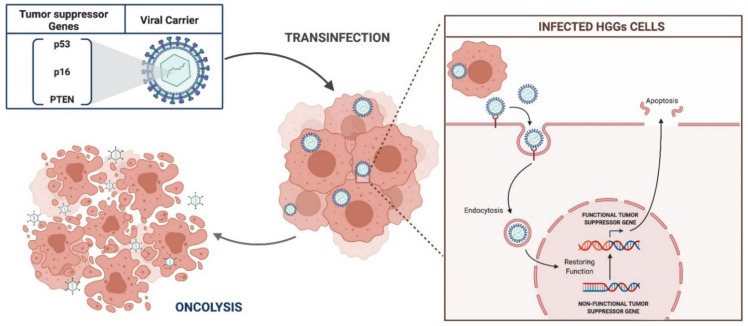
Schematic representation of tumor suppressor gene therapy.

Oncogenesis predicts the loss of the physiological regulatory function of some tumor suppressor genes which control the cell cycle and death. HGGs harbor deletions and mutations of specific tumor suppressors, more frequently the p53, p16, and phosphatase and tensin homologue (PTEN) [97]. The goal of tumor suppressor gene strategies is to transfer antitumoral functional genes to glioma cells, in order to restore normal function (Figure 4).

##### p53

The TP53 is the most common suppressor gene which codifies for p53 protein, fundamental in cell replication and apoptosis, found mutated in more than 50% of HGGs, 30% newly diagnosed, and 70% relapsed [98,99]. P53 is involved in angiogenesis inhibition and DNA repairing mechanisms.

The most accredited strategy includes the replication-deficient adenovirus in which the E1 gene is replaced by the wild-type p53 and conveyed by a cytomegalovirus promoter (Ad5CMV-p53). E1 deletion makes the virus unable to activate the infectious process, while the CMV promoter increases p53 gene expression [30,100,101]. Ad5CMV-p53 proved to block the glioma cell cycle, inhibit angiogenesis, and induce tumor apoptosis in many preclinical trials. [100,102,103,104,105,106]. In 1998, Badie et al. tested the efficacy of Ad-mediated p53 gene therapy, combined with radiation, in p53-mutant rat glioma models. Results showed 85% of tumor cell apoptosis in 24 h [107]. Later, Cirielli and his group studied the transfer of AdCMVp53 in human intracranial HGG cells in mice. 100 days after treatment all rats survived [103]. Another preclinical experimental study was conducted, in 2014, by Kim and colleagues. They designed a nanodelivery system able to carry the p53 gene into glioma cells through the blood-brain barrier. They reported a high rate of tumor suppression in GBM xenograft mice [108].

As regards clinical studies, two completed phase I trials employed the Ad5CMV-p53 as neo- and adjuvant therapy for relapsing HGGs. In both studies, patients received a preoperative intratumoral stereotactic inoculation of Ad5CMV-p53, followed by conventional surgery. Afterward, Ad5CMV-p53 was directly injected several times into the tumor cavity walls. Results showed a PFS of 13 weeks and OS of 44 weeks (#NCT00004041, #NCT00004080). A phase I clinical trial tested the efficacy and safety profile of Ad-p53 for HGGs treatment. 15 patients were enrolled and preoperatively treated with a stereotactic injection of Ad-p53 through an implanted catheter. After surgical gross total removal, Ad-p53 was injected several times in the surgical cavity. Treatment demonstrated low toxicity, but still limited efficacy [105].

##### p16

p16 controls the cell cycle arrest at the G1-S transition, avoiding uncontrolled replication and oncogenesis [109]. Restoration of p16 function, via adenoviral carrier, shown to inhibit glioma growth and locoregional diffusion, also blocking the activity of matrix metalloproteases in the glioma microenvironment [110]. In 1997 Chintala et al. tried to restore in vitro the p16 activity in HGGs cells, through Matrigel-coated transwell inserts and fetal rat-brain aggregates, by recombinant replication-deficient adenovirus. All tests showed a substantial reduction in glioma cell replication activity and a decrease in the expression of tumor microenvironment enzymes [110]. In 2000, Hung et al. tested the injection of a retrovirus, encoding the human p16 gene, in 10 rat HGG models. Results demonstrated the inhibition of glioma cell growth [111]. In 2003, Hama and colleagues examined the interaction between p16 and radiation-induced cell death. p16-null human glioma cell lines were induced to phase G1 of the cell cycle, by means of the adenovirus-mediated p16 gene. Data suggested that p16 expression is related to tumor radiosensitive via mechanisms of abnormal nucleation in HGG cells [112]. It is relevant that the effectiveness of the p16 gene strategy is only possible if the pRB activity is preserved [113].

##### PTEN

PTEN was found mutated in about 45% of HGGs and is involved in tumor microenvironment maintenance and proangiogenetic pathways [114,115]. Adenoviral delivery of the PTEN gene has been demonstrated to inhibit glioma proliferation and promote oncolysis [116,117,118,119]. In 1998, Cheney and colleagues designed a replication-defective adenovirus to transfer the PTEN gene in nude mice tumors. Results supported the tumor suppression activity of PTEN expression in HGGs [117]. Furthermore, as demonstrated by Davies and his group, PTEN inhibits Akt kinase activity, resulting in glioma cell death [116]. In vivo experiments, conducted by Abe and Lu, proved the adenoviral expression of PTEN able to block the angiogenetic processes and tumor proliferation in glioma cells [118,119]. In 2011, Inaba et al. demonstrated that the transmission of the PTEN gene into glioma cells, by an adenoviral vector, increased the tumor sensitivity to temozolomide and radiotherapy [120]. Table 6 presents the clinical trials on tumor suppressor gene therapies for HGGs.

#### 3.2.4. Immunomodulatory Gene Therapies

**Figure 5:**
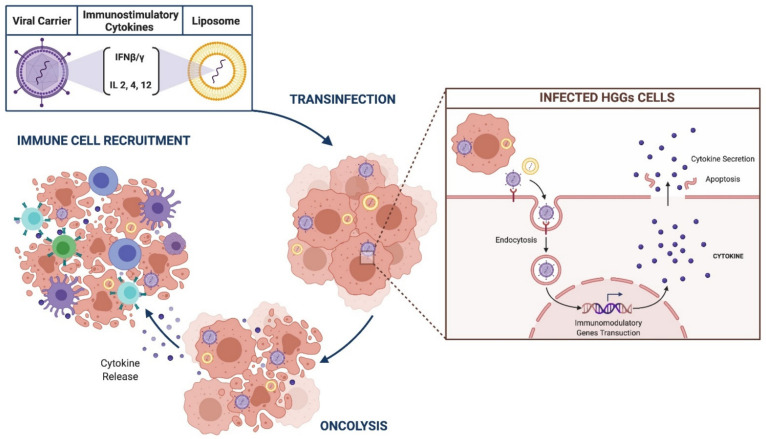
Schematic representation of immunomodulatory gene therapy.

HGGs resistance to standard treatments resides in the immune-escape tumor mechanisms and immunosuppressor tumor microenvironment. Immunomodulatory gene strategies are designed to implement the immune response against glioma by means of delivery of genes which encode for immunostimulatory cytokines and IFNβ/γ [48,85,121,122]. (Figure 5).

##### IFN-β/γ

Adenoviral-IFN-β gene delivery was tested in many preclinical and clinical trials [123,124,125,126,127,128,129]. In 2001, Qin et al. employed adenovirus expressing IFN-β in both in vivo and ex vivo human glioma xenografts in mouses. Results showed a potential antitumoral activity with the activation of NK cells and macrophages [123]. In phase I clinical study the drug was stereotactically inoculated into glioma before surgery. Results supported the activation of immune cascade and T and NK cells recruitment in the tumor microenvironment (#NCT00031083).

Nanoparticles and liposomes were also employed for INF- β transfer. From 1999, Natsume et al. conducted in vivo experiments using murine INF-β gene directly injected via liposomes in brain gliomas in mice. Results showed in 40% of cases the total inhibition of glioma growth with a strong antitumoral T lymphocyte infiltration [124]. Moreover, the same group carried on another study with the aim to deepen the role of tumor-specific lymphocytes. Mice were re-treated with a subcutaneous or intracranial injection of glioma cells and no tumor evidence was found 50 days later. This data proved that, in addition to the anticancer effects of INF-β, the local immune response has a role in long-term antitumor efficacy [125]. In 2004, Yoshida and colleagues tested the treatment with liposome/INF-β in a clinical trial, involving five patients with HGG. Four of these experienced a total or partial response to treatment with radiological evidence of volumetric glioma reduction by 50% and concomitant low toxicity [126]. Histological findings reported a high level of immune activation, also [127].

IFN-γ has the role of reducing cancer cell proliferation and interaction with the extracellular matrix [128]. IFN-γ as monotherapy was proved to be less effective, so combination protocols are under evaluation [129]. In 2002, Ehtesham and colleagues tested the efficacy of adenoviral-mediated IFN-γ and TNF-α gene transfer in HGGs cells. They proved the antitumoral efficacy of this treatment in mice models, also highlighting local increased recruitment of lymphocytes [130].

Furthermore, parvoviruses were engineered as vehicles of IFN-γ inducible protein 10 (CXCL10) and TNF-α, showing a synergic effect in tumor regression in rats HGGs models [131].

##### IL12/4/2

Among the immunostimulant cytokines, IL12 has a paramount role in boosting the immune cascade and recruiting cytotoxic lymphocytes at the tumor microenvironment [132,133,134].

Earlier phase studies employed non-replicating adenoviruses and HSV for delivery of IL12 to malignant glioma cells [135,136,137]. In 2012, Chiu et al. tested the intracranial injection of recombinant adeno-associated virus expressing IL12 gene (rAAV2/IL12) in the glioma mice model [135]. Later that year, Markert et al. studied the efficacy and security of γ34.5-deleted HSV1, encoding the IL12 gene, for malignant glioma treatment in rats [137]. Results of both preclinical studies showed tumor cell apoptosis, infiltration of active microglia cells, good safety profile, and strong local immune reaction.

Two recruiting phase I clinical trials tested the inducible adenoviral vector engineered to express IL12 (Ad-RTS- hIL12) with the oral vedelimex (an IL12 immunotherapeutic activator) for adult and pediatric gliomas (#NCT02026271, #NCT03330197). These studies revealed an intense upregulation of antitumor infiltrating lymphocytes.

IL4, secreted by lymphocytes, upregulates the immune cascade and B and T cells enrollment [138,139,140]. In clinical and preclinical models, the IL4 gene was virally transduced as an immunomodulatory agent for HGGs treatment. Yu and colleagues experienced the antitumoral activity of IL4, which was administered to 12 nude mice affected by gliomas. The treatment resulted in significant inhibition of glioma cell growth [138]. Okada and his group conducted clinical studies to test a vaccine constituted of IL-4-HSV-TK gene-modified autologous glioma cells, followed by systemic ganciclovir administration. Patients enrolled harbored recurrent/refractory supratentorial malignant glioma. The aim was to evaluate the safety profile, clinical efficacy, and immune response. Results reported good antitumoral activity and a strong antitumoral peripheral immunization [140].

Moreover, in 2005, Colombo et al. tested the intratumoral injection of retroviruses expressing both HSV-TK and IL2 genes, followed by intravenous ganciclovir, for treatment of 12 patients with recurrent HGGs. Few side effects were reported, and the 12 months PFS and OS was of 14% and 35%, respectively [141].

Table 7 summarizes the clinical trials on immunomodulatory gene therapies for HGGs.

#### 3.2.5. Gene Target Therapies

**Figure 6:**
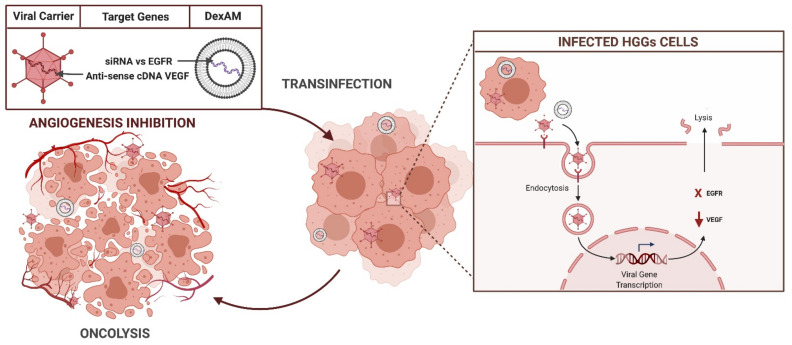
Target gene mechanism of action.

The identification of specific molecular markers of HGGs allowed the development of gene target therapies, designed to directly bind specific tumor antigens, with the aim to irreversibly block oncogenic pathways. Most of these strategies are still experimental and no active clinical trials are underway (Figure 6).

##### EGFRvIII

EGFRvIII variant, found in 30% of HGGs, is involved in mechanisms of oncogenesis and tumor progression [142,143]. Viral vectors and nanoparticles were engineered to transfer antisense or small interfering RNA directed specifically against the TK domain of glioma EGFRvIII. Several studies demonstrated a significant tumor volume reduction after treatment [144,145,146,147]. In 2006, Kang and colleagues projected antisense-RNA and small interference RNA (siRNA) expressing antisense EGFR genes which selectively bind the TK domain of EGFRvIII. After inoculation, glioma cell growth was amply reduced in vitro and in vivo models [145]. Shir and Levitzki tested antisense-RNA, transduced via viral and non-viral carriers, able to activate dependent protein kinase PKR. PKR induces cancer cell death targeting EGFRvIII in intracranial glioma xenografts [144]. In 2005, Padfield et al. confirmed the role of adjuvant miRNA therapies as promising strategies in glioma treatment. In fact, miR-7 showed high efficacy in blocking directly the EGFR pathways and downregulate MAPK/PI3K/Akt signaling, resulting in tumor cell apoptosis [148]. The cyclodextrin-modified dendritic polyamine complexes (DexAMs) were employed in the delivery of EGFRvIII siRNA and showed promising results in malignant glioma cells, also in combination with erlotinib [149].

##### VEGF/VEGFR

The vascular endothelial growth factor (VEGF) was found overexpressed in many malignant tumors. Adenoviral vector, loaded with anti-sense cDNA VEGF (Ad5CMV-αVEGF), was subcutaneously injected in nude mice previously infected with human glioma cells, resulting in inhibition of tumor spreading [150]. In xenografts, the direct intratumoral inoculation of PEI/VEGF siRNA showed an antiangiogenetic strong effect [151]. In 2007, Yoo and colleagues tested an oncolytic adenovirus (Ad)-based short hairpin RNA (shRNA) expression system (Ad-DeltaB7-shVEGF) directed versus the VEGF. Ad-DeltaB7-shVEGF showed high antiangiogenetic activity in the matrigel plug assay, and greater bioavailability compared to replication-incompetent adenoviruses [152]. The oncolytic adenovirus Ad-DeltaB7, was also employed in a preclinical study by Kang et al. in 2008. They designed an adenovirus able to express the transcriptional repressor Cys2-His2 zinc-finger proteins, F435-KOX, directed versus the VEGF promoter (Ad-DeltaB7-KOX). Ad-DeltaB7-KOX demonstrated high antitumor activity in a human xenografted glioma model [153].

In addition, the strategy of antagonizing the VEGF receptor (VEGFR) has proven to be effective. In 2004, Heidenreich et al. tried to inhibit the VEGFR-2 signaling pathway through transfers of a mutant-VEGFR via a retrovirus. The lack of intracellular tyrosine kinase domain in the engineered mutant-VEGFR resulted in inhibition of angiogenesis and progression in the xenografted glioma model [154]. A further study tested the coinfection of HGGs with adenovirus expressing VEGFR and an oncolytic virus dl922/947. This combined treatment resulted in more effectiveness than monotherapy [155].

## 4. Discussion

The present literature review aims to outline the up-to-date gene therapies for HGGs treatment, focusing especially on the molecular mechanisms, vectors, and therapeutic genes employed.

The rationale of gene strategies lies in the reprogramming of the glioma genome, intending to induce oncolysis or the expression of the antitumoral mediators. Manufactured genes are transferred to target cells through specific carriers, engineered to selectively bind cancer cells. Viral carriers were the first vehicle used, because of their specific neurotropism, and proved their gene delivery efficacy [156]. The main limitations are the short bioavailability of viral carrier and the negligible permanence of the virus at the tumor side [157].

Combined complex of viral vehicles with immunomodulatory agents is currently under investigation to enhance the duration of the therapeutic effect [158]. Among non-viral vehicles, only the liposomes were approved to be tested in clinical trials showing low toxicity and a high biodistribution level. All other nanoparticles are still in earlier phase studies.

If viral carriers are the most suitable vehicles for gene therapy, also the oncolytic virotherapy proved to be a valuable option. OVs act as a genetic payload which directly lyses tumor cells. The apoptosis of cancer cells promotes the release of tumor-associated antigens (TAA) in the tumor microenvironment. TAA are recognized by immune cell, resulting in the burst of immune cascade [159,160,161].

Suicide gene therapy is also an excellent potential resource for HGGs treatment. This approach is based on the assumption that suicide enzymes are not expressed in healthy cells. Therefore, the intravenous administration of prodrug and the intratumoral inoculation of virus-mediated suicide genes allow restricting the therapeutic effect only to glioma cells, while reducing systemic side effects [76,82,162]. Another considerable advantage of suicide gene therapy lies in the “bystander effect”, namely the ability to share transduced genes and death signals to the neighboring cells through gap junctions [163,164,165].

In the era of translational medicine, the identification of specific tumor markers and genes involved in oncogenesis, above all EGFR, VEGF, TP53, and pRB pathways, offers new insights to design the target gene strategies and tumor suppressor gene therapies [22]. These lasts are based on the rearrangement of the glioma genome with the aim of restoring lost oncosuppressive functions. Delivery of tumor suppressor genes can be exploited as a combined approach, resulting in sensibilization of glioma cells to chemoradiotherapy [106,166].

Despite good assumptions, the intrinsic heterogeneity of HGGs, the multitude of mutations, and immune evasion mechanisms constitute the major limits of all these strategies. The immunomodulatory gene therapies, including INF e cytokines delivery, were projected precisely to modulate the immunosuppressive tumor microenvironment, meanwhile increasing the oncolytic gene therapy efficacy [134].

In accordance with the data outlined in the present review, the most accredited strategies are oncolytic virotherapy (26 trials), suicide gene strategies (18 trials), and immunomodulatory gene therapies (14 trials). Results reported overall excellent effectiveness, especially as adjuvant therapies with local injection after surgery. No significant toxicity was reported and, when preoperatively administered, a role in reducing tumor volume was also demonstrated.

The future perspectives of the HGGs treatment are directed toward the progressive integration of standard chemoradiotherapy with immune-boosting strategies and new tailored gene therapies.

## 5. Conclusions

Gene therapies are projected with the aim to edit the glioma genome and overcome the therapeutic resilience of HGGs. The oncolytic virotherapy, suicide genes and immunomodulatory strategies, tumor suppressor, and target genes therapies were widely tested in clinical trials, remaining mostly still experimental approaches.

Oncolytic viruses oHSVs and CRAds were proven to be safe and feasible. HSV-TK and CD suicide genes revealed a promising potential in several preclinical studies. Although they are not included in the first-line treatment protocol for newly diagnosed HGGs, gene therapies represent a valuable option as second-line adjuvant therapy for refractory GBM.

Future perspectives provide for the development of new administration vehicles, optimize biodistribution and selectivity. Further clinical trials are essential to implement standard protocols with gene innovative strategies in therapeutic synergy.

## Figures and Tables

**Table 1:** Inclusion and Exclusion Criteria for Literature Review.

Inclusion Criteria	Exclusion Criteria
Reviews, Peer-Reviews, Editorials	Case Reports, Abstracts, and Dissertations
Clinical, Pre-clinical Trials	Abandoned Clinical Trials
English language, or translated	Non-English language
Publications in 2005–2020 decade	Studies prior to 2005
Studies on Human, or Human Products	Animal Studies
Neuro-oncology relevance	Publications not related to neuro-oncology
Publications about High-Grade Glioma treatment	Publications not related to High-Grade Glioma

**Table 2:** Comparison between viral and non-viral vectors.

	Vectors				
	Viral			Non-Viral	
	AD	HSV	RT	Cationic Liposomes	Polymers (PEI, PAMAM)
Diameter (nm)	150–200	100–300	100	20–200	50–250
Genetic Payload	dsDNA	dsDNA	RNA	dsDNA/RNA	dsDNA/RNA
Transduction Efficiency	High	Very High	Medium	High	High
Immunogenicity	Very High	Very High	Medium	None	None
Mutagenesis Risk	None	None	Yes	None	None

AD: Adenovirus; HSV: Herpes Simplex Virus; PAMAM: Poly-Amidoamine Polymer; PEI: Polyethylenimine; RT: Retrovirus.

**Table 3:** Classification of Gene Therapies for High-Grade Gliomas.

Gene Therapies		
Oncolytic Virotherapy	Oncolytic viruses	oHSV
		CRAd
		MV
		PVS-RIPO
Suicide Gene Therapy	Suicide Genes	TK
		CD
		PNP
Tumor Suppressor Gene Therapy	Tumor Suppressor Genes	p53
		p16
		PTEN
Immunomodulatory Gene Therapy	Immunomodulatory Genes	IFNβ/γ
		IL-4, IL-12
Gene Target Therapy	Target Genes	EGFRvIII
		VEGF

CD: Cytosine Deaminase; CRAd: Conditionally Replicating Adenovirus; EGFRvIII: Epidermal Growth Factor Receptor Variant vIII; oHSV: Oncolytic Herpes Simplex Virus; IFN: Human Interferon; IL: Interleukine; MV: Measles Paramyxovirus; PNP: Purine Nucleoside Phosphorylase; PTEN: Phosphatase and Tensin Homologue; PVS-RIPO: Recombinant Nonpathogenic Polio-Rhinovirus; TK: Thymidine Kinase; VEGF: Vascular Endothelial Growth Factor.

**Table 4:** Clinical trials on oncolytic virotherapy for high-grade gliomas.

#	ClinicalTrials.gov Identifier	Title	Status	Phase	Diseases	# of Pts. Enrolled	Treatment	Locations
1	NCT00028158	Safety and Effectiveness Study of G207, a Tumor-Killing Virus, in Patients with Recurrent Brain Cancer	Completed	I/II	Glioma Astrocytoma Glioblastoma	65	Drug: G207, an oncolytic virus	NA
2	NCT00157703	G207 Followed by Radiation Therapy in Malignant Glioma	Completed	I	Malignant Glioma	9	Drug: G207, an oncolytic virus	USA
3	NCT02031965	Oncolytic HSV-1716 in Treating Younger Patients with Refractory or Recurrent High-Grade Glioma That Can Be Removed by Surgery	Terminated	I	Brain and Central Nervous System Tumors	2	Biological: oncolytic HSV-1716; Drug: dexamethasone Procedure: therapeutic conventional surgery	USA
4	NCT03152318	A Study of the Treatment of Recurrent Malignant Glioma With rQNestin34.5 v.2	Recruiting	I	Brain and Central Nervous System Tumors	108	Drug: rQNestin, Cyclophosphamide; Procedure: Stereotactic biopsy	USA
5	NCT02197169	DNX-2401 With Interferon Gamma (IFN-γ) for Recurrent Glioblastoma or Gliosarcoma Brain Tumors	Completed	I	Glioblastoma Gliosarcoma	37	Single intratumoral injection of DNX-2401; Drug: Interferon-gamma	USA
6	NCT01174537	New Castle Disease Virus (NDV) in Glioblastoma Multiforme (GBM), Sarcoma and Neuroblastoma	Withdrawn	I/II	Glioblastoma Sarcoma Neuroblastoma	0	Biological: New Castle Disease Virus	IL
7	NCT00390299	Viral Therapy in Treating Patients With Recurrent Glioblastoma Multiforme	Completed	I	Anaplastic Astrocytoma Anaplastic Oligodendroglioma Mixed Glioma Recurrent Glioblastoma	23	Biological: Carcinoembryonic Antigen-Expressing Measles Virus; Therapeutic Conventional Surgery	USA
8	NCT01301430	Parvovirus H-1 (ParvOryx) in Patients with Progressive Primary or Recurrent Glioblastoma Multiforme.	Completed	I/II	Glioblastoma Multiforme	18	Drug: H-1PV	DE
9	NCT01582516	Safety Study of Replication-competent Adenovirus (Delta-24-rgd) in Patients with Recurrent Glioblastoma	Completed	I/II	Brain Tumor Recurring Glioblastoma	20	Biological: delta-24-RGD adenovirus	NL
10	NCT02062827	Genetically Engineered HSV-1 Phase 1 Study for the Treatment of Recurrent Malignant Glioma	Recruiting	I	Recurrent Glioblastoma Multiforme Progressive Glioblastoma Multiforme Anaplastic Astrocytoma or Gliosarcoma	36	Biological: M032 (NSC 733972)	USA
11	NCT03911388	HSV G207 in Children with Recurrent or Refractory Cerebellar Brain Tumors	Recruiting	I	Brain and Central Nervous System Tumors	15	Biological: G207	USA
12	NCT00805376	DNX-2401 (Formerly Known as Delta-24-RGD-4C) for Recurrent Malignant Gliomas	Completed	I	Brain Cancer Central Nervous System Diseases	37	Drug: DNX-2401 Procedure: Tumor Removal	USA
13	NCT03896568	Oncolytic Adenovirus DNX-2401 in Treating Patients with Recurrent High-Grade Glioma	Recruiting	I	Brain and Central Nervous System Tumors	36	Oncolytic Adenovirus Ad5-DNX-2401 Therapeutic Conventional Surgery	USA
14	NCT01956734	Virus DNX2401 and Temozolomide in Recurrent Glioblastoma	Completed	I	Glioblastoma Multiforme Recurrent Tumor	31	Procedure: DNX2401 and Temozolomide	ES
15	NCT02986178	PVSRIPO in Recurrent Malignant Glioma	Active, not recruiting	II	Malignant Glioma	122	PVSRIPO	USA
16	NCT03973879	Combination of PVSRIPO and Atezolizumab for Adults with Recurrent Malignant Glioma	Withdrawn	I/II	Malignant Glioma	0	Biological: PVSRIPO Drug: Atezolizumab	NA
17	NCT03043391	Phase 1b Study PVSRIPO for Recurrent Malignant Glioma in Children	Recruiting	I	Brain and Central Nervous System Tumors	12	Biological: Polio/Rhinovirus Recombinant (PVSRIPO)	USA
18	NCT01491893	PVSRIPO for Recurrent Glioblastoma (GBM)	Active, not recruiting	I	Glioma Malignant Glioma	61	Recombinant nonpathogenic polio-rhinovirus chimera (PVSRIPO)	USA
19	NCT03072134	Neural Stem Cell Based Virotherapy of Newly Diagnosed Malignant Glioma	Active, not recruiting	I	Brain and Central Nervous System Tumors	NA	Neural stem cells loaded with an oncolytic adenovirus	NA
20	NCT03657576	Trial of C134 in Patients with Recurrent GBM	Active, not recruiting	I	Glioblastoma Multiforme of Brain Anaplastic Astrocytoma of Brain Gliosarcoma of Brain	24	Biological: C134	USA
21	NCT02798406	Combination Adenovirus + Pembrolizumab to Trigger Immune Virus Effects	Active, not recruiting	II	Brain and Central Nervous System Tumors	49	Biological: DNX-2401 Biological: pembrolizumab	USA
22	NCT03714334	DNX-2440 Oncolytic Adenovirus for Recurrent Glioblastoma	Recruiting	I	Glioblastoma Glioblastoma, Adult	24	Drug: DNX-2440 injection	ES
23	NCT03294486	Safety and Efficacy of the Oncolytic Virus Armed for Local Chemotherapy, TG6002/5-FC, in Recurrent Glioblastoma Patients	Recruiting	I/II	Glioblastoma Brain Cancer	78	Drug: Combination of TG6002 and 5-flucytosine (5-FC, Ancotil^®^)	FR
24	NCT02457845	HSV G207 Alone or With a Single Radiation Dose in Children With Progressive or Recurrent Supratentorial Brain Tumors	Active, not recruiting	I	Brain and Central Nervous System Tumors	12	Biological: G207	USA
25	NCT00006106	ONYX-015 With Cisplatin and Fluorouracil in Treating Patients with Advanced Head and Neck Cancer	Withdrawn	I	Lip and Oral Cavity Cancer Head and Neck Cancer Oropharyngeal Cancer	0	Drug: Cisplatin, Fluorouracil Drug: ONYX-015	USA
26	NCT00528684	Safety and Efficacy Study of REOLYSIN^®^ in the Treatment of Recurrent Malignant Gliomas	Completed	I	Malignant Glioma	18	Biological: REOLYSIN^®^	USA

DE: Germany; ES: Spain; FR: France; GBM: Glioblastoma Multiforme; HSV: Herpes Simplex Virus; IL: Israel; IFN-γ: Interferon Gamma; NDV: New Castle Disease Virus; NL: Netherlands; Pts: Patients; PVSRIPO: Recombinant Nonpathogenic Poliorhinovirus; USA: United States of America.

**Table 5:** Clinical trials on suicide gene therapies for high-grade gliomas.

#	ClinicalTrials.gov Identifier	Title	Status	Phase	Diseases	# of Pts. Enrolled	Treatment	Locations
1	NCT00870181	ADV-TK Improves Outcome of Recurrent High-Grade Glioma	Completed	II	Malignant Glioma of Brain Glioblastoma	47	Biological: ADV-TK/GCV Procedure: Surgery Drug: systemic chemotherapy	CHN
2	NCT00002824	Gene Therapy in Treating Patients with Primary Brain Tumors	Completed	I	Brain and Central Nervous System Tumors	NA	Biological: gene therapy Drug: chemotherapy, ganciclovir Procedure: conventional surgery	USA
3	NCT00751270	Phase 1b Study of AdV-tk + Valacyclovir CombinedWith Radiation Therapy for Malignant Gliomas	Completed	I	Malignant Glioma Glioblastoma Multiforme Anaplastic Astrocytoma	15	Biological: AdV-tk Drug: Valacyclovir	USA
4	NCT03596086	HSV-tk + Valacyclovir + SBRT + Chemotherapy for Recurrent GBM	Recruiting	I/II	Glioblastoma Multiforme Astrocytoma, Grade III	62	Drug: ADV/HSV-tk (gene therapy)	USA
5	NCT00634231	A Phase I Study of AdV-tk + Prodrug Therapy in Combination with Radiation Therapy for Pediatric Brain Tumors	Active, not recruiting	I	Malignant Glioma Recurrent Ependymoma	12	Biological: AdV-tk Drug: valacyclovir Radiation: Radiation	USA
6	NCT00589875	Phase 2a Study of AdV-tk with Standard Radiation Therapy for Malignant Glioma (BrTK02)	Completed	II	Malignant Glioma Glioblastoma Multiforme Anaplastic Astrocytoma	52	Biological: AdV-tk Drug: Valacyclovir	USA
7	NCT03603405	HSV-tk and XRT and Chemotherapy for Newly Diagnosed GBM	Recruiting	I/II	Glioblastoma Anaplastic Astrocytoma	62	Drug: ADV/HSV-tk (gene therapy)	USA
8	NCT00001328	Gene Therapy for the Treatment of Brain Tumors	Completed	I	Brain Neoplasm Neoplasm Metastasis	15	Drug: Cytovene (Ganciclovir Sodium) Device: G1TKSVNa.53 Producer Cell Line	USA
9	NCT03576612	GMCI, Nivolumab, and Radiation Therapy in Treating Patients with Newly Diagnosed High-Grade Gliomas	Active, not recruiting	I	Glioma, Malignant	36	Biological: AdV-tk, Nivolumab Drug: Valacyclovir, Temozolomide; Radiation	USA
10	NCT01985256	Study of a Retroviral Replicating Vector Given Intravenously to Patients Undergoing Surgery for Recurrent Brain Tumor	Completed	I	Glioblastoma Multiforme Anaplastic Astrocytoma Anaplastic OligodendrogliomaAnaplastic Oligoastrocytoma	17	Biological: Toca 511 Drug: Toca FC	USA
11	NCT01156584	A Study of a Retroviral Replicating Vector Combined with a Prodrug Administered to Patients with Recurrent Malignant Glioma	Completed	I	Glioblastoma Anaplastic Astrocytoma Anaplastic OligodendrogliomaAnaplastic Oligoastrocytoma	54	Biological: Toca 511Drug: Toca FC	USA
12	NCT01174537	New Castle Disease Virus (NDV) in Glioblastoma Multiforme (GBM), Sarcoma and Neuroblastoma	Withdrawn	I/II	Glioblastoma Sarcoma Neuroblastoma	0	Biological: New Castle Disease Virus	IL
13	NCT01470794	Study of a Retroviral Replicating Vector Combined with a Prodrug to Treat Patients Undergoing Surgery for a Recurrent Malignant Brain Tumor	Completed	I	Glioblastoma Multiforme Anaplastic Astrocytoma Anaplastic OligodendrogliomaAnaplastic Oligoastrocytoma	58	Biological: Toca 511 Drug: Toca FC	USA
14	NCT00390299	Viral Therapy in Treating Patients with Recurrent Glioblastoma Multiforme	Completed	I	Anaplastic Astrocytoma Anaplastic Oligodendroglioma Mixed Glioma Recurrent Glioblastoma	23	Biological: Carcinoembryonic Antigen-Expressing Measles Virus; Therapeutic Conventional Surgery	USA
15	NCT02414165	The Toca 5 Trial: Toca 511 & Toca FC Versus Standard of Carec in Patients with Recurrent High-Grade Glioma	Terminated	II/III	Glioblastoma Multiforme Anaplastic Astrocytoma	403	Biological: Toca 511, Bevacizumab; Drug: Toca FC; Drug: Lomustine, Temozolomide	USA
16	NCT01811992	Combined Cytotoxic and Immune-Stimulatory Therapy for Glioma	Active, not recruiting	I	Malignant Glioma Glioblastoma Multiforme	19	Dose Escalation of Ad-hCMV-TK and Ad-hCMV-Flt3L	USA
17	NCT02598011	A Study of the Safety of Toca 511, a Retroviral Replicating Vector, Combined with Toca FC in Subjects with Newly Diagnosed High Grade Glioma Receiving Standard of Care	Withdrawn	I	Newly Diagnosed High Grade Glioma (HGG)	0	Biological: Toca 511 Drug: Toca FC	NA
18	NCT04406272	VB-111 in Surgically Accessible Recurrent/Progressive GBM	Recruiting	II	Glioblastoma Recurrent Glioblastoma	45	Drug: VB11 Procedure: Surgery Drug: Bevacizumab	USA

CHN: China; CMV: Citomegalovirus; GBM: Glioblastoma Multiforme; GCV: Ganciclovir; IL: Israel; NA: Not Available; Pts: Patients; XRT: Radiotherapy.

**Table 6:** Clinical trials on tumor suppressor gene therapies for high-grade gliomas.

#	ClinicalTrials.gov Identifier	Title	Status	Phase	Diseases	# of Pts. Enrolled	Treatment	Locations
1	NCT00004041	Gene Therapy in Treating Patients with Recurrent Malignant Gliomas	Completed	I	Brain and Central Nervous System Tumors	NA	Biological: Ad5CMV-p53 gene; Procedure: conventional surgery	USA
2	NCT00004080	Gene Therapy in Treating Patients with Recurrent or Progressive Brain Tumors	Completed	I	Brain and Central Nervous System Tumors	NA	Biological: recombinant adenovirus-p53 SCH-58500; Procedure: conventional surgery	NA

Ad: Adenovirus; CMV: Citomegalovirus; NA: Not Available; Pts: Patients; USA: United States of America.

**Table 7:** Clinical trials on immunomodulatory gene therapies for high-grade gliomas.

#	ClinicalTrials.gov Identifier	Title	Status	Phase	Diseases	# of Pts. Enrolled	Treatment	Locations
1	NCT00031083	Dose Escalation Study to Determine the Safety of IFN-Beta Gene Transfer in the Treatment of Grade III & Grade IV Gliomas	Completed	I	Glioblastoma Multiforme Anaplastic Astrocytoma Oligoastrocytoma, Mixed Gliosarcoma	12	Genetic: Interferon-beta	USA
2	NCT02026271	A Study of Ad-RTS-hIL-12 With Veledimex in Subjects with Glioblastoma or Malignant Glioma	Active, not recruiting	I	Glioblastoma Multiforme Anaplastic Oligoastrocytoma	48	Biological: Ad-RTS-hIL-12; Drug: veledimex	USA
3	NCT03679754	Evaluation of Ad-RTS-hIL-12 + Veledimex in Subjects with Recurrent or Progressive Glioblastoma, a Substudy to ATI001-102	Active, not recruiting	I	Glioblastoma Multiforme	36	Biological: Ad-RTS-hIL-12; Drug: veledimex	USA
4	NCT03636477	A Study of Ad-RTS-hIL-12 With Veledimex in Combination With Nivolumab in Subjects with Glioblastoma; a Substudy to ATI001-102	Active, not recruiting	I	Glioblastoma Multiforme	21	Biological: Ad-RTS-hIL-12 Drug: veledimexDrug: Nivolumab	USA
5	NCT03330197	A Study of Ad-RTS-hIL-12 + Veledimex in Pediatric Subjects with Brain Tumors Including DIPG	Recruiting	I/II	Pediatric Brain Tumor Diffuse Intrinsic Pontine Glioma	45	Biological: Ad-RTS-hIL-12 Oral Veledimex	USA
6	NCT03866109	A Study Evaluating Temferon in Patients with Glioblastoma & Unmethylated MGMT	Recruiting	I/II	Glioblastoma Multiforme	21	Temferon	IT
7	NCT03383978	Intracranial Injection of NK-92/5.28. z Cells in Patients with Recurrent HER2-positive Glioblastoma	Recruiting	I	Glioblastoma Multiforme	30	Biological: NK-92/5.28.z	DE
8	NCT04165941	Novel Gamma-Delta (γδ)T Cell Therapy for Treatment of Patients With Newly Diagnosed Glioblastoma	Recruiting	I	Brain Tumor Adult	12	Biological: DRI cell therapy	USA
9	NCT04214392	Chimeric Antigen Receptor (CAR) T Cells with a Chlorotoxin Tumor- Targeting Domain for the Treatment of MPP2 + Recurrent or Progressive Glioblastoma	Recruiting	I	Recurrent Glioblastoma Recurrent Malignant Glioma Recurrent WHO Grade II Glioma Recurrent WHO Grade III Glioma	36	Biological: Chlorotoxin (EQ)-CD28-CD3zeta-CD19t- expressing CAR T-lymphocytes	USA
10	NCT02208362	Genetically Modified T-cells in Treating Patients with Recurrent or Refractory Malignant Glioma	Recruiting	I	Brain and Central Nervous System Tumors	92	IL13Ralpha2-specific Hinge-optimized 4-1BB-co-stimulatory CAR/Truncated CD19-expressing Autologous TN/MEM Cells; IL13Ralpha2-specific Hinge-optimized 41BB-co-stimulatory CAR Truncated CD19-expressing Autologous T-Lymphocytes	USA
11	NCT00730613	Cellular Adoptive Immunotherapy Using Genetically Modified T-Lymphocytes in Treating Patients with Recurrent or Refractory High-Grade Malignant Glioma	Completed	I	Brain and Central Nervous System Tumors	3	Biological: therapeutic autologous lymphocytes Genetic: gene expression analysis	NA
12	NCT00005796	Combination Chemotherapy Plus Gene Therapy in Treating Patients with CNS Tumors	Completed	I	Bone Marrow Suppression Brain and Central Nervous System Tumors	10	Filgrastim, gene therapy, lomustine; procarbazine, vincristine sulfate	USA
13	NCT02444546	Wild-Type Reovirus in Combination with Sargramostim in Treating Younger Patients with High-Grade Relapsed or Refractory Brain Tumors	Active, not recruiting	I	Brain and Central Nervous System Tumors	6	Biological: Sargramostim Biological: Wild-type Reovirus	USA
14	NCT01082926	Phase I Study of Cellular Immunotherapy for Recurrent/Refractory Malignant Glioma Using Intratumoral Infusions of GRm13Z40-2, An Allogeneic CD8 + Cytolitic T-Cell Line Genetically Modified to Express the IL 13-Zetakine and HyTK and to be Resistant to Glucocorticoids, in Combination with Interleukin-2	Completed	I	Brain and Central Nervous System Tumors	6	Biological: therapeutic allogeneic lymphocytes; Biological: aldesleukin	USA

Ad-RTS-hIL-12: Inducible Adenoviral Vector Engineered to express IL-12; CAR: Chimeric Antigen Receptor; CNS: Central Nervous System; DE: Germany; DIPG: Diffuse Intrinsic Pontine Glioma; GBM: Glioblastoma Multiforme; HyTK: Hybromycin Thymidine Kinase; IFN: Interferon; MGMT: 0-6-Methylguanine DNA-methyltransferase; MPP2: Palmitoylated membrane protein 2; NK: Natural Killer; Pts: Patients; WHO: World Health Organisation.

## Data Availability

All data are included in the main text.
